# The Role of Zinc and Zinc Homeostasis in Macrophage Function

**DOI:** 10.1155/2018/6872621

**Published:** 2018-12-06

**Authors:** Hong Gao, Wei Dai, Lu Zhao, Junxia Min, Fudi Wang

**Affiliations:** The First Affiliated Hospital, School of Public Health, Institute of Translational Medicine, Zhejiang University School of Medicine, Hangzhou 310058, China

## Abstract

Zinc has long been recognized as an essential trace element, playing roles in the growth and development of all living organisms. In recent decades, zinc homeostasis was also found to be important for the innate immune system, especially for maintaining the function of macrophages. It is now generally accepted that dysregulated zinc homeostasis in macrophages causes impaired phagocytosis and an abnormal inflammatory response. However, many questions remain with respect to the mechanisms that underlie these processes, particularly at the cellular and molecular levels. Here, we review our current understanding of the roles that zinc and zinc transporters play in regulating macrophage function.

## 1. Introduction

A healthy human body usually contains 2–4 grams of zinc [1]. Approximately 60% of the body's zinc is located in the skeletal muscle, 30% in the bone, 5% in the liver and the skin, and the remaining 2–3% in other tissues [2]. Internal zinc homeostasis is regulated by the cooperative activities of two metal transporter protein families. One family consists of ten solute-linked carrier 30 (SLC30 or ZnT) exporters, and the other family consists of fourteen solute-linked carrier 39 (SLC39, also known as Zrt- and Irt-like proteins, or ZIP) importers [3, 4]. The majority of labile zinc in the body is absorbed by intestinal epithelial cells via the metal transporter protein Slc39a4 [5], which is then transported into the plasma and utilized by nearly all cell types in the circulation. To maintain zinc homeostasis, excessive zinc is excreted through the kidneys [6] and the intestine [7] via Slc39a5.

Endogenous zinc is usually present in two forms in various organs and tissues. The majority of zinc is in a fixed pool in which zinc is tightly bound to metalloenzymes and zinc finger transcription factors; the remaining small amount of zinc is in a labile pool consisting of a variable amount of loosely bound zinc and free zinc ions [8]. In mammals, the plasma concentration of zinc ranges from 14 to 23 *μ*mol/l under normal physiological conditions, and serum zinc accounts for only 0.1% of the body's total zinc pool, 80% loosely bound by albumin and 20% bound by macroglobulin [9, 10]. Thus, sufficient daily intake of zinc is required to achieve steady-state levels. In order to meet the daily requirement, the World Health Organization recommends a daily zinc intake of 9.4–10 mg and 6.5–7.1 mg for men and women, respectively [11].

Zinc plays an important role in the immune system and affects both innate and adaptive immune cells. Many studies found that zinc deficiency can lead to a reduced immune response and increased susceptibility to infection [12–16]. Moreover, endogenous zinc levels have been suggested to affect both the number and the function of various types of immune cells, including macrophages, neutrophils, dendritic cells, mast cells, T cells, and B cells [17–24]. The underlying molecular mechanisms have been discussed in previous studies [25, 26], and the importance of zinc as a signaling molecule has been suggested [17, 27].

Macrophages play a key role in innate immunity by regulating numerous homeostatic, developmental, and host defense responses. Moreover, macrophages also participate in a wide range of other biological activities, including modulating endogenous levels of reactive oxygen species [28, 29], iron homeostasis [30], tissue repair, and metabolic processes [31]. Macrophages have three major functions—phagocytosis, antigen presentation, and immunomodulation—and are essential for maintaining normal immune status under a wide variety of pathophysiological conditions [32]. Many previous studies investigated the relationship between zinc and macrophages [33–37]; however, some studies yielded contradictory results, and the underlying mechanisms are poorly understood. Here, we provide an overview of the latest studies regarding the role of zinc in macrophages.

## 2. Zinc Homeostasis in Macrophages

The regulation of zinc homeostasis is a complicated process. As a divalent cation, zinc is hydrophilic and does not readily pass lipid-based cell membranes via passive diffusion; thus, specialized transporters are required in order to facilitate its transport in and out of the cytoplasm. In macrophages and many other immune cells, SLC39 and SLC30 family members have distinct expression patterns and have various functions in response to infectious stimuli (Table 1).

Multiple SLC30/SLC39 members are expressed in macrophages. In untreated mouse macrophages, *Slc39a1*, *Slc39a6*, and *Slc39a7* are the most robustly expressed genes in the Slc39a family, whereas the *Slc30a5*, *Slc30a6*, *Slc30a7*, and *Slc30a9* genes are the most robustly expressed genes in the Slc30a family [22], suggesting that these transporters play an important role in macrophages under physiological conditions. However, under pathological conditions, other key transporters are expressed. For example, upon stimulation with lipopolysaccharides (LPS), which are found in the outer membrane of gram-negative bacteria, *Slc39a10* expression is significantly downregulated, whereas *Slc39a14* expression is strongly upregulated [22, 38]. Moreover, Slc39a2, Slc30a4, and Slc30a7 are significantly upregulated in GM-CSF-activated peritoneal and bone marrow-derived macrophages [39].

Several SLC30/39 members have been found to participate in the function of macrophages by mediating zinc homeostasis. Our recent study using macrophage-specific *Slc39a10*-knockout mice revealed that Slc39a10 plays an essential role in p53-dependent macrophage survival following LPS stimulation [22]. Interestingly, the trans-fatty acid elaidate was found to increase the expression of *SLC39A10* and increase intracellular zinc levels in human macrophages [40], which also indicates the importance of Slc39a10 in zinc homeostasis in macrophages. In addition, several studies reported that SLC39A8 plays a role in inflammatory reactions [41, 42]. For example, LPS has been suggested to upregulate the expression of *SLC39A8* in human macrophages, thereby increasing zinc uptake and reducing proinflammatory pathways by inhibiting I*κβ* kinase (IKK) [41] and IL-10 [42]. Furthermore, SLC39A14 was also found to be upregulated in response to LPS stimulation in macrophages, thereby regulating cytokine production [38]. Moreover, systemic inflammation in mice resulted in the IL-6-dependent upregulation of the zinc importer Slc39a14, which mediates zinc uptake by hepatocytes in the liver [43]. Although previous studies summarized above suggest functions of Slc39a8, Slc39a10, and Slc39a14 in macrophages, potential roles of other SLC39/30 transporters in macrophages [22, 44–48] remain to be explored.

Recently, a growing body of evidence supports the notion that zinc transporters transport not only zinc but also other divalent metals, including iron and manganese; for example, both SLC39A8 and SLC39A14 have been associated with iron and manganese transport [49–55]. These findings raise the question of whether other SLC30/39 family members are involved in the development and functions of macrophage through mediating the homeostasis of other metals, such as iron or manganese.

In addition to the two zinc transporter families, intracellular zinc levels are also regulated by metallothioneins (MTs). Because of its toxicity, intracellular labile zinc is generally present in extremely low levels. Laurin et al. reported that adding zinc to the culture medium increased the rate of MT degradation and decreased the rate of MT synthesis and accretion in a chicken macrophage cell line [56].

Several groups reported that MTs play a role in macrophage function. MT-I/II-knockout mice developed more severe brain injury accompanied by increased numbers of T cells in the injury site and circulating leukocytes and the decreased number of alternatively activated macrophages in the circulation after 7-day treatment with brain cryolesion. These observations indicate that MT-I/II may have a neuroprotective role via modulation of the immune response [57]. Besides, Zbinden et al. measured increased numbers of macrophages in the ischemic hind limb of MT-deficient mice 21 days after ischemia was induced; moreover, CD11b+ macrophages isolated from MT-deficient mice were more invasive, which indicates that MT plays an important role in the recovery of collateral flow and angiogenesis, an effect mediated partly by macrophages [58]. In addition, in *Salmonella typhimurium*-infected human monocyte-derived macrophages, NOD2 mediates the induction of MT via NF-*κ*B- and caspase-1-mediated IL-1*β* secretion. Moreover, the elevated MT level was found to upregulate intracellular zinc in a MTF-1-dependent manner. However, the underlying mechanism remains unclear [59]. Furthermore, during alternative activation of macrophages, IL-4 increases intracellular zinc dependence on metallothionein-3 (MT-3) and Slc30a4 and weakens the antimicrobial defense against intracellular pathogens [60]. In addition, matrix metallothionein 7 (MMP7) cleaves the precursor forms of *α*-defensin and *β*-defensin to produce their respective active forms [61], and MMP12 destroys the pathogen's cell wall, leading to cell death [62]. In summary, a wide range of MTs are involved in maintaining macrophage function during the immune response.

## 3. Zinc and the Macrophage Cell Fate

Zinc homeostasis determines the cell fate of macrophages. In the innate immune system, monocytes migrate into the infected tissue and then differentiate into macrophages. Zinc supplementation increases the number of peritoneal macrophages in a *T. cruzi* infection model [63]. In addition, zinc-depleted monocytes have increased maturation, suggesting that low zinc status promotes their differentiation into macrophages [64]. High concentrations of zinc were found to decrease the viability of a human monocyte cell line and U-937 cells [65]. Moreover, another study confirmed that cell viability is significantly decreased in THP-1 monocytes/macrophages upon exposure to 100 *μ*g/ml of ZnO (zinc oxide) particles. However, ZnO nanoparticles were found to induce the migration, adhesion, and cholesterol uptake of monocytes/macrophages, which may accelerate the formation of foam cells and lead to atherosclerosis [66]. Furthermore, a low-zinc environment can inhibit the differentiation of HL-60 cells into macrophages, and this inhibition can be partially prevented by the addition of exogenous zinc [67]. As in other cell types, both zinc deficiency and excessive zinc can induce apoptosis in macrophages. For example, using a genetic mouse model, we recently found that loss of Slc39a10 reduces zinc levels in macrophages, resulting in p53-dependent apoptosis, but not necroptosis, pyroptosis, ferroptosis, or autophagy [22]. On the other hand, zinc oxide nanoparticles have been shown to induce necrosis and apoptosis in RAW264.7 cells [68–70]. These results suggest that altered zinc homeostasis induces distinct forms of cell death under different circumstances.

## 4. Zinc and Macrophage Function

Innate immunity provides a rapid, nonspecific defense against pathogens and is activated by pathogen-associated molecular patterns (PAMPs). During this process, conserved structures in pathogens are recognized by their respective receptors, including Toll-like receptors (TLRs), which then trigger phagocytosis, cytokine secretion, the killing of target cells, and/or antigen presentation [71]. Monocytes/macrophages mediate host defense via phagocytosis and oxidative burst. In addition, these cells can serve as antigen-presenting cells (APCs) and can secrete proinflammatory cytokines in order to regulate the immune response [72, 73]. Zinc plays a critical role in the immune function of macrophages, and this function has been implicated in a variety of pathological processes, including decreased connective tissue contraction [34].

### 4.1. Zinc and Phagocytosis by Macrophages

The level of intracellular zinc influences the phagocytosis capacity of macrophages, and zinc was recently linked to the antimicrobial response in macrophages [33]. In chronic obstructive pulmonary disease (COPD), impaired efferocytosis (i.e., clearance) of apoptotic epithelial cells by alveolar macrophages is mediated primarily by zinc restriction [44]. The transporters Slc39a1 and Slc39a2 respond differently to zinc deficiency and play important roles in macrophage-mediated efferocytosis [44]. On the other hand, zinc does not affect the phagocytic function of RAW264.7 cells [74] or bone marrow-derived macrophages [22] at nontoxic concentrations. Interestingly, a recent study by Mehta et al. found that alcohol abuse is associated with significant zinc deficiency in alveolar macrophages, which is accompanied by impaired immune function due to decreased phagocytosis-mediated bacterial clearance [75]. The authors also found that treating alveolar macrophages with zinc significantly improved their phagocytic capacity [75]. An earlier study by Wirth et al. found that zinc deficiency impairs the uptake and survival of protozoan parasites [76]. Zinc supplementation was also found to increase the phagocytosis of *E. coli* and *Staphylococcus aureus* by peritoneal macrophages in a mouse model of polymicrobial sepsis. Notably, Sheikh et al. reported that zinc deficiency decreases the phagocytic capacity of monocytes in children with enterotoxigenic *E. coli*-induced diarrhea, whereas treating patients with zinc (20 mg/day) or dietary zinc supplementation (10 mg/day) slightly improved the monocytes' phagocytic capacity and significantly decreased their cellular oxidative burst capacity [77]. From a clinical perspective, these effects of zinc supplementation with respect to alleviating symptoms in zinc-deficient children are highly encouraging.

### 4.2. Zinc and Oxidative Burst in Macrophages

The relationship between zinc and the level of oxidative burst in macrophages after bacterial infection is controversial. Mayer et al. reduced zinc concentrations in peripheral blood mononuclear cells—which include monocytes—either by treating the cells with TPEN (N,N,N′,N′-tetrakis(2-pyridylmethyl)-ethylenediamine) or by removing zinc from the culture medium using the chelator Chelex 100. They found that the level of oxidative burst was significantly increased in zinc-deficient macrophages following infection with gram-positive *S. aureus* [73]. In addition, zinc is an inhibitor of NADPH, which is the electron donor for catalyzing the production of O_2_^−^ [78]. On the other hand, Srinivas et al. found that macrophages obtained from *E. coli*-infected rats released significantly higher amounts of superoxide and that *in vivo* superoxide production was increased by zinc supplementation; nevertheless, they also found that zinc supplementation *in vitro* inhibited the production of superoxide by macrophages harvested from septic rats [79].

### 4.3. Zinc and Inflammatory Signaling in Macrophages

Zinc also plays essential roles in the signaling and inflammatory output of monocytes and macrophages, including many upstream activators of the Toll-like receptor (TLR) family, including mitogen-activated protein kinase (MAPK), protein kinase C (PKC), phosphodiesterases, and NF-*κ*B [36, 37]. Indeed, the relationship between zinc and inflammatory signaling in monocytes/macrophages relies primarily on TLR signaling (e.g., via TLR4), which is activated by the phosphorylation of interleukin-1 receptor-associated kinase 1 (IRAK1). Zinc is known to be required for the degradation of IRAK1 in LPS-stimulated TLR activation both *in vitro* and *in vivo*; however, zinc is not required for the phosphorylation or ubiquitylation of IRAK1 in macrophages [80]. Nevertheless, zinc has been found to mediate the degradation of procaspase-1 and the NLRP3 (NLR family, pyrin domain containing 3), as well as to inhibit the production of IL-1*β* in macrophages following LPS stimulation or *Salmonella* infection. This effect may compromise the cell's ability to clear microbial pathogens [45].

TLR4 signaling occurs via MyD88-dependent and TRIF-dependent pathways, and zinc has opposing effects on these two signaling pathways. Upon LPS stimulation, TLR4 first binds to the adapter proteins TIRAP and MyD88, which triggers the phosphorylation of MAP kinases and the early activation of NF-*κ*B. Zinc signaling is required for preventing the dephosphorylation of the MAP kinases p38, MEK1/2, and ERK1/2, as well as the activation of NF-*κ*B. Thus, zinc increases the release of inflammatory cytokines such as TNF-*α*, IL-1*β*, and IL-6 [81, 82]. Subsequently, the receptor complex is internalized and binds to TRAM and TRIF, inducing the delayed activation of NF-*κ*B and the phosphorylation of IRF3. Phosphorylated IRF3 then translocated to the nucleus, where it induces the transcription of IFN-*β* [82, 83]. However, zinc can inhibit the phosphorylation of IRF3 and can prevent the secretion of IFN-*β* [82]. Moreover, zinc supplementation could downregulate inflammatory cytokines through upregulation of A20 to inhibit NF-*κ*B activation [78, 84].

Zinc deficiency has diverse effects on inflammation. Zinc deficiency over the long term reduces the integrity of lysosomes, activates the NLRP3 inflammasome, and induces IL-*1β* secretion in macrophages [85], while in the short term, zinc depletion by TPEN inhibits inflammatory activation [86]. Moreover, without adequate zinc, an inflammatory response can also be elicited in cells, in part by causing the aberrant activation of immune cells and/or by altering promoter methylation [87]. In addition, a recent study found that zinc deficiency reduces the production of IL-6 and TNF-*α* in human monocytes [73]. Finally, zinc modulates LPS-induced inflammation in human macrophages by inducing SLC39A8 and by inhibiting C/EBP*β* [42].

ZnO nanoparticles also affect the innate immune process. For example, ZnO nanoparticles have been shown to reduce bacterial skin infection by inducing oxidative stress and causing cell membrane breakdown in macrophages [88], as well as by reducing the innate immune response and attenuating the macrophage responses to bacterial infection [89]. In contrast, ZnO nanoparticles have been shown to induce a proinflammatory response in the RAW264.7 macrophage cell line [66, 90] and in peritoneal macrophages via TLR6-mediated MAPK signaling [91]. These seemingly contradictory results may be due—at least in part—to the different concentrations of nanoparticles and/or cell types used in the different studies.

Taken together, the evidence to date suggests that zinc regulates the function of macrophages in a variety of ways. For example, zinc deficiency induces the abnormal secretion of immune factors via distinct pathways in response to specific infections. In addition, oxidative stress caused by altered levels of zinc can lead to dysfunction of the innate immune system during acute inflammation.

## 5. Zinc and Macrophage-Related Diseases

According to a 2002 report by the World Health Organization, zinc deficiency ranks fifth among the most important health risk factors in developing countries and eleventh worldwide [92]; moreover, abnormal zinc homeostasis causes a variety of health problems with various levels of severity. In addition to the immune system, other organs and systems can also be affected by changes in zinc.

### 5.1. Immunological Diseases

The relationship between zinc and rheumatoid arthritis (RA) has been studied for more than three decades. RA is a chronic systemic inflammatory disease characterized by inflammation of the synovial membrane and the progressive destruction of the articular cartilage and bone [93]. Importantly, the number and activation level of macrophages in the inflamed synovial membrane/pannus are correlated with the severity of RA. A recent meta-analysis of 1444 RA cases and 1241 healthy controls revealed that patients with RA often have decreased serum zinc levels [94]. Correspondingly, the mean level of zinc was significantly lower in hair samples of RA patients compared with healthy individuals [95]. These clinical observations are supported by *in vitro* studies. For example, zinc deficiency increases the levels of TNF-*α*, IL-1*β*, and IL-8 in a monocyte-macrophage cell line [96]. In contrast, zinc supplementation inhibits the LPS-induced release of TNF-*α* and IL-1*β* in monocytes [97].

Chronic alcoholism can increase the risk of pneumonia and the development of acute respiratory distress syndrome (ARDS) [98]. As the resident bona fide phagocytic cell type in the lungs, alveolar macrophages play a central role in maintaining alveolar homeostasis, lung host defense, and immune regulation [99]. Several groups have studied the relationship between zinc levels and macrophage function in the alveolar space. For example, Mehta et al. found that alcohol-fed rats have a 5-fold decrease in lung bacterial clearance compared to control-fed rats and providing dietary zinc supplementation to the alcohol-fed rats restored bacterial clearance and mitigated oxidative stress in the alveolar space, which was reflected by the relative balance between the thiol redox pair cysteine and cystine and by the increased nuclear binding of both PU.1 and Nrf2 in alveolar macrophages obtained from alcohol-fed rats [90, 100]. Similarly, Konomi et al. found that during pregnancy, intracellular zinc levels and the expression levels of the zinc transporters Zip1, ZnT1, and ZnT4 are decreased in alveolar macrophages after ethanol ingestion compared to control rats that did not ingest alcohol. In addition, bacterial clearance capacity was decreased in ethanol-treated alveolar macrophages, and the addition of zinc reversed these effects *in vitro* [101]. Furthermore, pulmonary zinc deficiency may be one of the mechanisms by which HIV-1 infection impairs alveolar macrophage immune function and renders infected individuals susceptible to severe pulmonary infection [102].

### 5.2. Nonimmunological Diseases

Evidence suggested that chronic inflammation that originated in the liver or adipose tissue plays an important role in the pathogenesis of obesity-related metabolic dysfunction [103]. In obese mice, zinc deficiency may increase leptin production and stimulate macrophage infiltration into the adipose tissue, suggesting that zinc is important in metabolic and macrophage-mediated inflammatory dysregulation in obesity [104]. Based on its anti-inflammatory and antioxidant functions, zinc also plays a protective role in atherosclerosis [105]. However, zinc deficiency does not appear to affect the uptake of low-density lipoprotein (LDL) by macrophages *in vitro* [106]. Interestingly, another study found that ZnO nanoparticles can induce the migration and adhesion of monocytes to endothelial cells and accelerate the formation of foam cells [107].

### 5.3. Pathogen Infection

A sufficient amount of zinc is essential for the host's defense against pathogenic organisms. For example, in both human monocyte-derived macrophages and mouse macrophages, increased intracellular zinc levels induced by the continuous stimulation of pattern recognition receptors (PRRs) can increase the clearance of bacteria via autophagy [59]. Moreover, treating mice with zinc and/or all-*trans* retinoic acid supplements helps protect against infection by the pathogen *Listeria monocytogenes* [108].

Interestingly, zinc is not only required by host cells but is also required for invading pathogens. According to the “nutritional immunity” theory, specific essential elements are sequestered from pathogens in order to restrict their growth [109, 110]. Zinc chelation was shown to restrict the growth of certain pathogens, for example, *Histoplasma capsulatum* [64]. A previous study found that zinc deprivation may be a defense mechanism utilized by the host's macrophages [35]. Moreover, when stimulated with granulocyte macrophage-colony stimulating factor (GM-CSF), macrophages infected with *Histoplasma capsulatum* sequester zinc by inducing zinc binding to metallothionein (MT) proteins [39]. In addition, human macrophages attack intracellular *Mycobacterium tuberculosis* pathogens by inducing a “burst of labile zinc” and by increasing the expression of the zinc-binding proteins MT1, MT2, and ZnT1 [111], as well as possibly releasing zinc stored in zincosomes [112]. Macrophages can also use a “zinc trap” [113] to kill pathogens; this mechanism may be impaired when intracellular zinc is either too high or too low.

## 6. Conclusions and Future Perspectives

**Figure 1:**
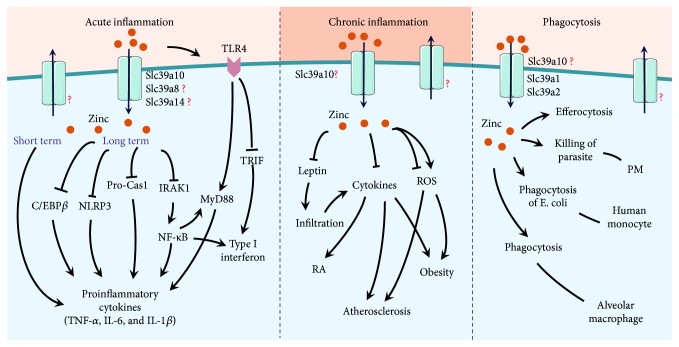
Schematic model depicting the putative roles that zinc plays in macrophages during acute inflammation, chronic inflammation, and phagocytosis. BMDM: bone marrow-derived macrophage; PM: peritoneal macrophage; RA: rheumatoid arthritis; ROS: reactive oxygen species; TRIF: Toll/IL-1R domain-containing adapter inducing IFN-*β*.

The vital role that the micronutrient zinc plays in both health and disease has been known for many years. Regular intake of zinc and the coordinated function of zinc transporters are essential for maintaining zinc homeostasis and for maintaining health. With respect to innate immunity, the various functions of macrophages, which include phagocytosis and the secretion of immune-mediating factors, can be impaired by zinc imbalance, thereby inducing or exacerbating various inflammatory and/or disease processes, as illustrated in Figure 1.

Despite extensive research, the molecular mechanisms by which zinc regulates the fate and function of macrophages remain poorly understood. Similarly, the function of zinc transporters is largely uninvestigated. In some cases, particularly when accompanied by a defect in a zinc transporter, oral zinc supplementation or restriction may not be sufficient for preventing diseases caused by cellular zinc imbalance; therefore, molecular approaches are needed in order to develop innovative new therapeutic approaches to correct the underlying defect. Given the development of powerful gene editing tools, the genetic manipulation of zinc transporters can be performed in various model systems, and research based on these models will likely shed light on the molecular function of these zinc transporters, as well as the mechanism of zinc in macrophages, ultimately guiding the treatment and prevention of zinc-related diseases.

## Figures and Tables

**(a):** 

Importer proteins	Expression in macrophages	Expression in other immune cells	Infection-related findings
Slc39a1	Strong expression in the plasma membrane and cytoplasm in THP1-derived macrophages [44]	Expressed in murine T cells [114]	HIV-1 stimulated Slc39a1 expression in alveolar macrophages [115]
Slc39a2	THP1 macrophages: weak expression mainly in nucleoli; TPEN significantly increases Slc39a2 expression Alveolar macrophages: strong expression in the plasma membrane and cytoplasm [44]	No expression in human monocytes or in granulocytes [46]; moderated expression in murine DCs [116]	Unknown
Slc39a3	Strong expression in human monocytes [46]	Expressed in human T cells and granulocytes [46]	Unknown
Slc39a4	Expressed in alveolar macrophages [117]	Uniform expression in human monocytes and in granulocytes [46]	Chronic alcohol exposure decreases Slc39a4 expression in alveolar macrophages [117]
Slc39a5	Unknown	No expression in human monocytes or in granulocytes [46]	Unknown
Slc39a6	Strong expression in murine macrophages [22]	Expressed in human DCs and T cells [20]	LPS decreases the expression of Slc39a6 in human DCs; Slc39a6-silenced macrophages have increased TNF*α* expression following LPS stimulation [20]
Slc39a7	Strong expression in murine macrophages [22], which can be inhibited by TPEN [45]	Expressed in murine T cells [114]	Unknown
Slc39a8	Strong expression in both human and murine macrophages	Strong expression in human T cells [21]	Both TNF*α* and LPS upregulate Slc39a8 expression in human macrophages, which increases zinc uptake and directly inhibits IKK*β* [41] and IL-10 [42]
Slc39a9	Unknown	Expressed in murine T cells [114]	Unknown
Slc39a10	Strong expression in murine macrophages	Expressed in murine early B cells [23] and T cells [114]	Slc39a10^fl/fl^; LysMCre+ mice have significantly decreased LPS-induced mortality due to increased macrophage apoptosis mediated by zinc-p53 signaling [22]
Slc39a11	Unknown	Expressed in murine T cells [114]	Unknown
Slc39a12	Unknown	Expressed in murine T cells, expression is increased by zinc deficiency [114]	Unknown
Slc39a13	Unknown	Unknown	Unknown
Slc39a14	Expressed in alveolar macrophages, expression is decreased by TPEN [44]	Expressed in leukocytes; Slc39a14-knockout mice have delayed leukocytosis [118]	LPS upregulates Slc39a14 expression and downregulates NF-*κ*B in human macrophages [38]; Slc39a14-knockout mice have impaired zinc uptake and decreased plasma zinc and IL-6 levels following LPS stimulation [119]

**(b):** 

Exporter proteins	Expression in macrophages	Expression in other immune cells	Infection-related findings
Slc30a1	Expressed in alveolar macrophages, expression is decreased by TPEN [44]	Expressed in murine DCs, expression is upregulated by LPS [18]	*M. tuberculosis* infection upregulates Slc30a1 expression in human macrophages [111]
Slc30a2	Weak expression in macrophages in the nulliparous mammary gland [47]; increased expression in murine macrophages during infection [39]	No expression in human monocytes or granulocytes [46]	Unknown
Slc30a3	Expressed in alveolar macrophages, expression is decreased by TPEN [44]	Expressed at low levels in human peripheral blood lymphocytes [48]	Unknown
Slc30a4	Unknown	Expressed in murine DCs, expression is upregulated by LPS [18]; highly expressed in the human Molt-4 T cell line [48]	GM-CSF upregulate Slc30a4 expression to transport zinc into Golgi [39]
Slc30a5	Expressed in alveolar macrophages, expression is decreased by TPEN [44]	Expressed in murine mast cells and required for the mast cell-mediated delayed-type allergic response [19]	Unknown
Slc30a6	Expressed in THP-1 monocytes, expression is upregulated by zinc deficiency [48]	Expressed in murine DCs, expression is upregulated by LPS [18]	Unknown
Slc30a7	Expressed in THP-2 monocyte, expression is upregulated by zinc deficiency [48]	Expressed in human B lymphocytes with the target molecule CD40 [120]	GM-CSF upregulates Slc30a7 expression, leading to increased zinc transport into the Golgi apparatus [39]
Slc30a8	Unknown	Expressed in human peripheral blood lymphocytes [48]	May function as an autoantigen targeted by disease-associated autoreactive T cells in humans [121]
Slc30a9	Strong expression in murine macrophages [22]	Expressed at low levels in human circulating blood lymphocytes [48]; expressed in murine T cells, expression is decreased by zinc deficiency [114]	Unknown
Slc30a10	Unknown	Unknown	Unknown

DCs: dendritic cells; GM-CSF: granulocyte-macrophage colony-stimulating factor; IL: interleukin; LPS: lipopolysaccharides; TPEN: N,N,N′,N′-tetrakis(2-pyridylmethyl)-ethylenediamine (a membrane-permeable zinc chelator).
